# Using a Bayesian Network Predictive Model to Understand Vulnerability of Australian Sheep Producers to a Foot and Mouth Disease Outbreak

**DOI:** 10.3389/fvets.2021.668679

**Published:** 2021-06-11

**Authors:** Jennifer Manyweathers, Yiheyis Maru, Lynne Hayes, Barton Loechel, Heleen Kruger, Aditi Mankad, Gang Xie, Rob Woodgate, Marta Hernandez-Jover

**Affiliations:** ^1^Graham Centre for Agricultural Innovation, Charles Sturt University, Wagga Wagga, NSW, Australia; ^2^School of Animal and Veterinary Sciences, Charles Sturt University, Wagga Wagga, NSW, Australia; ^3^Commonwealth Scientific and Industrial Research Organisation Land and Water, Canberra, ACT, Australia; ^4^Commonwealth Scientific and Industrial Research Organisation, Brisbane, QLD, Australia; ^5^Australian Bureau of Agricultural and Resource Economics and Sciences (ABARES), Canberra, ACT, Australia; ^6^Quantitative Consulting Unit, Charles Sturt University, Wagga Wagga, NSW, Australia

**Keywords:** Bayesian network model, foot and mouth disease, biosecurity, vulnerability, Australian sheep producers, surveillance, partnership

## Abstract

To maintain and strengthen Australia's competitive international advantage in sheep meat and wool markets, the biosecurity systems that support these industries need to be robust and effective. These systems, strengthened by jurisdictional and livestock industry investments, can also be enhanced by a deeper understanding of individual producer risk of exposure to animal diseases and capacity to respond to these risks. This observational study developed a Vulnerability framework, built from current data from Australian sheep producers around behaviors and beliefs that may impact on their likelihood of Exposure and Response Capacity (willingness and ability to respond) to an emergency animal disease (EAD). Using foot and mouth disease (FMD) as a model, a cross-sectional survey gathered information on sheep producers' demographics, and their practices and beliefs around animal health management and biosecurity. Using the Vulnerability framework, a Bayesian Network (BN) model was developed as a first attempt to develop a decision making tool to inform risk based surveillance resource allocation. Populated by the data from 448 completed questionnaires, the BN model was analyzed to investigate relationships between variables and develop producer Vulnerability profiles. Respondents reported high levels of implementation of biosecurity practices that impact the likelihood of exposure to an EAD, such as the use of appropriate animal movement documentation (75.4%) and isolation of incoming stock (64.9%). However, adoption of other practices relating to feral animal control and biosecurity protocols for visitors were limited. Respondents reported a high uptake of Response Capacity practices, including identifying themselves as responsible for observing (94.6%), reporting unusual signs of disease in their animals (91.0%) and daily/weekly inspection of animals (90.0%). The BN analysis identified six Vulnerability typologies, with three levels of Exposure (high, moderate, low) and two levels of Response Capacity (high, low), as described by producer demographics and practices. The most influential Exposure variables on producer Vulnerability included adoption levels of visitor biosecurity and visitor access protocols. Findings from this study can guide decisions around resource allocation to improve Australia's readiness for EAD incursion and strengthen the country's biosecurity system.

## Introduction

The Australian sheep industry has long been recognized for its significant contribution to the global sheep meat and wool industries (1). Protecting the sheep meat and wool industries in the face of increasing global risks associated with significant disease outbreaks such as foot and mouth disease (FMD) (2–4) and maintaining Australia's clean and green reputation should remain a high priority. The current animal health surveillance system for notifiable diseases includes targeted programs but also relies on general surveillance primarily underpinned by producers notifying their private or government veterinarian of unusual signs of disease. By strengthening the capacity of rural communities and the producers themselves to rapidly identify and contain any possible future emergency animal disease (EAD) outbreaks will enhance preparedness of the existing surveillance systems (2, 5–7).

Historically, Australian Government resources have facilitated locally based veterinarians and supported personnel spending time on-farm. In keeping with global trends (8–10), these resources are decreasing, resulting in a weakening of local relationships and diminishing the strong surveillance networks that previously existed (2, 11–13). The “shared responsibility” approach to surveillance adopted by the Australian Government (4) has great potential. However, in practice, it has not been met with a consistent level of engagement by stakeholders (10). This has resulted in a perception among producers that the government is devolving itself of responsibility for surveillance and decreasing the priority of livestock industries more generally, further undermining the surveillance system (2, 14).

Any weakening of partnerships within the surveillance system is of particular concern within the sheep industry, which has traditionally had lower levels of engagement with animal health professionals (15, 16). Learnings from the FMD outbreak across the United Kingdom suggest that sheep are likely to play a significant role in any undetected occurrence and spread of FMD in the event of an outbreak because of the variable and transient nature of ovine FMD symptoms (6, 17, 18). As part of maintaining and strengthening protective biosecurity and surveillance strategies, and strengthening partnerships, understanding the diversity of the sheep industry and its unique risk profile is vital.

Risk-based approaches to surveillance for FMD could reduce the probability of threats occurring and assuage consequences of a possible FMD outbreak in Australia. These approaches can assist the identification of potential routes of entry and establishment of FMD and effective allocation of resources for surveillance and response (2, 8). The foundation of such an approach is a comprehensive understanding of the factors that may influence both likelihood of exposure to FMD and the response capacity (willingness and ability) to respond to an outbreak. At a national level, East et al. (5) used a multicriteria analysis and found that current surveillance efforts are effectively targeting the highest risk areas for an FMD incursion. Subsequent research has highlighted further opportunities for improving FMD outbreak preparedness by decreasing the time between introduction and diagnosis (19). Garner et al. (20) also showed an inverse correlation between the likelihood of successful eradication of FMD and the time taken for initial diagnosis.

To reduce the time taken between infection and diagnosis, understanding biosecurity beliefs and practices of producers and those regularly in contact with livestock is paramount (12, 21). This includes the ability to recognize unusual signs of disease in livestock and to take appropriate steps to get a diagnosis (22). When modeling the spread of FMD from a single index farm, one of the most influential factors was found to be the ability of the producer to detect unusual signs leading to identification of FMD (23). Therefore, any risk-based approach to surveillance needs to be informed by stakeholder engagement, including producers' beliefs and practices around their role in general surveillance, defined in this study as monitoring for, recognizing, and reporting unusual signs of disease in their animals (22, 24–27).

To inform risk-based approaches to strengthening surveillance strategies, an EAD risk characterization of livestock producers based on FMD vulnerability was developed from a cross-sectional survey that collected producer information from the FMD-susceptible livestock industries in Australia. The survey data were then used to populate a Bayesian Network (BN) model for analysis of producer vulnerability (28). This paper focuses on Australian sheep producers' beliefs and practices that may influence their likelihood of exposure and capacity to respond to an FMD outbreak. The results pertaining to the Australian beef and goat industries have been reported elsewhere (22, 29).

## Materials and Methods

A cross-sectional study was designed, and a questionnaire was developed to gather quantitative data to build a vulnerability-based typology of the Australian sheep industry, using FMD as a model. The methodological approach used for this study was the same of that used in Manyweathers et al. (22, 29). All research activities were approved by the Human Research Ethics Committee at Charles Sturt University (H400201720).

### Questionnaire Design

**Figure 1:**
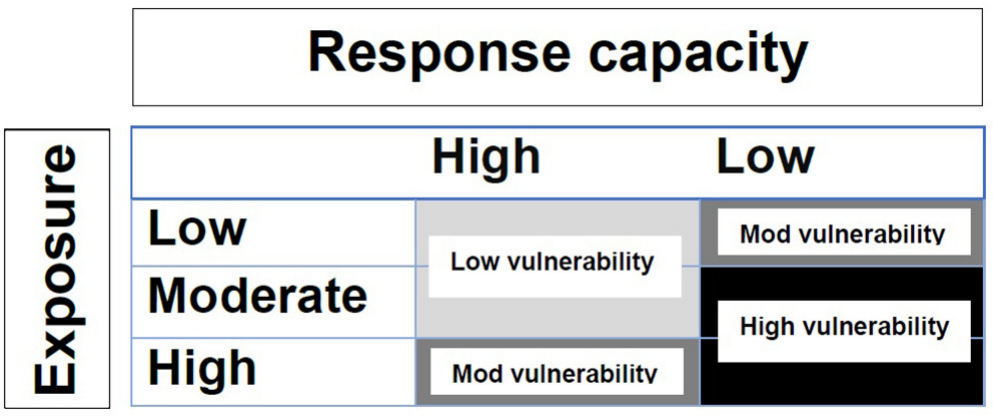
Classification matrix of vulnerability as the intersection of exposure and response capacity: light gray = low vulnerability; medium gray = moderate vulnerability; black = high vulnerability [(22), adapted from Nelson et al. (30)].

The questionnaire was developed with reference to existing epidemiological, behavioral, and social science research and aimed at the examination and characterization of sheep producers' vulnerability to an FMD outbreak. Further details of the design and development of the questionnaire are provided by Manyweathers et al. (22). In brief, a vulnerability matrix (Figure 1) guided questionnaire development, with questions examining producers' likelihood of exposure to FMD and their response capacity (willingness and ability to inspect animals, detect and report disease) to an FMD outbreak. The questionnaire was developed by a multidisciplinary team, including social and behavioral science researchers, veterinarians, systems science, and biosecurity researchers, through an iterative process. The questionnaire was then piloted prior to distribution by one sheep industry representative, one veterinarian, and two sheep producers, to improve validity and clarity.

The questionnaire, which consisted of 61 questions, gathered information in relation to practices, perceptions, and attitudes around the risk of and response to a FMD outbreak. The following four main areas were included in the questionnaire: *Demographics and husbandry practices* (24 questions), *Biosecurity practices and beliefs* (8), *Animal health management practices* (23), and *Networks and trust* (10).

### Questionnaire Distribution

The questionnaire was distributed online and via post between August 2017 and June 2018, with final distribution routes guided by available support from industry and government agencies. Multiple farming system groups from the Riverina region in New South Wales (NSW), along with Local Lands Services (NSW government agency), distributed the questionnaire via direct email link to members, with accompanying coverage in newsletters and on Twitter and Facebook. The Livestock Biosecurity Network distributed the questionnaire link in their newsletters and also discussed the study during biosecurity workshops in NSW. Interested producers were followed up and provided with the link. In addition, a link to the questionnaire was distributed by the Graham Centre for Agricultural Innovation, a research center of Charles Sturt University, and NSW Department of Primary Industries, as well as the Victorian Farmers Federation, via their newsletters. In Western Australia (WA), a random sample of 750 sheep producers with a registered Property Identification Code (PIC) was emailed a link to the questionnaire.

Sheep producers who completed the questionnaire were invited to enter a draw for 20 × $50 retail vouchers. In addition, they also had the opportunity to enter a draw for two smart tablets across participants from all industries.

### Data Analysis

#### Descriptive Analysis

Data from the online and postal questionnaires were collated in Excel (PC/Windows XP, 2007) and checked for data entry errors. Descriptive statistics were used to obtain an overview of participant demographic and husbandry characteristics, practices, and attitudes (IBM SPSS Statistics for Windows, Version 20.0. Armonk, NY: IBM Corp.).

#### Bayesian Network Analysis

A Bayesian network model is a probabilistic graphical tool that allows for modeling of biological, social, and physical systems that operate under uncertainty (28, 31) and is suitable for a large number of different data types and hidden variables being connected through complex relationships (32). Formally, a BN model is a graphical representation, i.e., a directed acyclic graph (DAG), of a joint probability distribution of a set of random variables in which each variable is represented by a node and the dependence relationship is represented by a link/edge for two associated variables (28, 33).

Essentially, a BN model follows a machine learning approach for data analysis. Although the theoretical foundation and computational algorithms underlying BNs are utilized in subjects such as computer science, mathematics, and statistics, the applications of BN models are very intuitive and relatively straightforward because of the availability of many well-tested BN application software packages (28, 31). In this study, we used the most popular commercial BN software Netica (version 6.05) (34) to characterize Australian sheep producers based on their enterprises' vulnerability to an FMD outbreak. This approach has also been used by beef and goat producers (22, 29).

**Figure 2:**
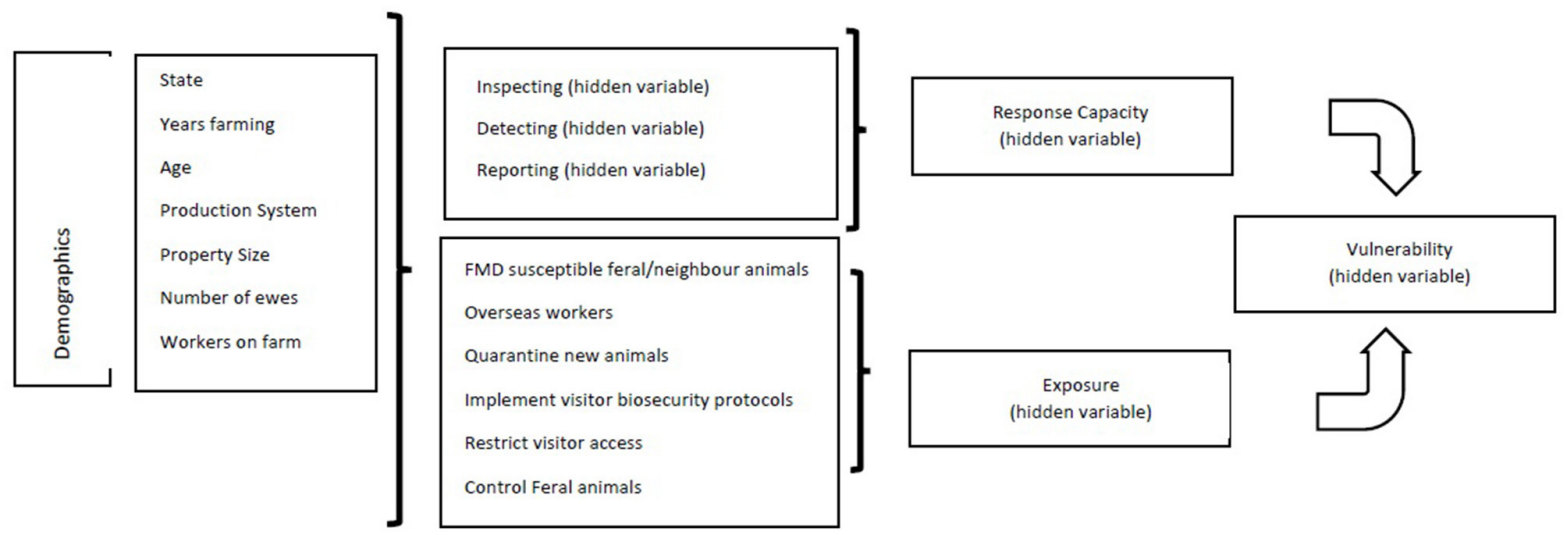
Bayesian network conceptual model for examining Australian sheep producers' vulnerability to foot and mouth disease (FMD).

Every BN model has two components in its model specification. The qualitative component of a BN specifies the network structure through a set of (conditional) dependence and independence statements among a set of random variables, informational precedence, and preference relations; the quantitative component of a BN determines the conditional probability tables (CPTs) that quantifies the strengths of dependence relations using probability theory and preference relations using utility theory (28). In this study, the BN model development processes started with the conceptual model as detailed in Figures 1, 2. The data collected from sheep farmers included 41 observed variables. According to our disciplinary theory, these observed variables were the building blocks for defining various composite or hidden variables with which the level of “vulnerability” could be determined. Table 1 specifies which hidden variable was defined/characterized by the observed variables and the layers of the hidden variables. The hierarchical structure of the hidden variables and how they related to those observed variables are shown in Figure 2. The top level hidden variable “vulnerability” was defined by “response capacity” and “exposure.” In turn, “response capacity” was further defined by three sublevel hidden variables, and the “exposure” was defined directly by seven observed variables; those demographics variables were all observed variables and should affect both “response capacity” and “exposure.” Note that, due to space limitation, there were a large number of observed variables behind the three sublevel hidden variables that are not displayed in Figure 2 with detailed information provided in Table 1.

**Table 1:** A list of questions considered for assessing the likelihood of exposure and response capacity of Australian sheep producers to an foot and mouth disease (FMD) outbreak and the classifications of response.

**Questions**				
**Exposure related questions**		**Classification of responses**		
		**High likelihood**	**Moderate likelihood**	**Low likelihood**
*Do you*				
Employ overseas workers?		Yes	Occasionally	No
Isolate new stock?		Never, rarely	Occasionally	Most of the time, always
Restrict access?		Never, rarely	Occasionally	Most of the time, always
Require visitor biosecurity practices?		Never, rarely	Occasionally	Most of the time, always
Take action to control feral animals?		Never, rarely	Yes	Most of the time, always
Have neighbors with FMD Susceptible species?		Yes	Yes	No
Have FMD susceptible feral species on your property?		Yes		No
**Response-capacity-related questions**		**Classification of responses**		
		**High capacity**	**Low capacity**	
How frequently do you undertake the following activities?				
	Visual inspection	Once a day, once a week	Once a month, few times a year, once a year or less, never	
	Visual and physical inspection	Once a day, once a week	Once a month, few times a year, once a year or less, never	
	Inspection of unwell animals	Once a day, once a week	Once a month, few times a year, once a year or less, never	
Who do you think is responsible for				
	Inspecting animals for unusual signs	Me, staff	Private or gov vet, stock agent, neighbors, industry group	
	Recognizing unusual signs of disease	Me, staff	Private or gov vet, stock agent, neighbors, industry group	
	Reporting unusual signs	Me, staff	Private or gov vet, stock agent, neighbors, industry group	
In the last 12 months, how often have you				
	Used an NVD/health statement when buying animals	Always, most of the time	Occasionally, rarely, never	
	Inspected stock before buying them	Always, most of the time	Occasionally, rarely, never	
How confident are you that you could identify FMD in your sheep				
		Extremely, very, moderately	Slightly, Not at all	
Rank first three actions when you see unusual signs of disease				
	Call private vet	1st, 2nd 3rd action	Not in top 3 actions	
	Call gov vet	1st, 2nd 3rd action	Not in top 3 actions	
	Watch and wait	Not in top 3 actions	1st, 2nd 3rd action	
	Do nothing	Not in top 3 actions	1st, 2nd, 3rd action	
	Call hotline	1st, 2nd, 3rd action	Not in top 3 actions	
In a single event, what number of animals showing unusual				
signs/dead would you be concerned about				
	Number showing unusual signs	<10	10–50, more than 50	
	Number animals dead	<5, 5–10	11–50, more than 50	
How often have you				
	Reported unusual signs	Always, most of the time	Occasionally, rarely, never	
Do you use				
	Private vets	Yes	No	
	Govt vets	Yes	No	
Do you trust				
	Private vets	Completely, very, moderately	A little, not at all	
	Govt vets	Completely, very, moderately	A little, not at all	

Initially, the conceptual model specified that the categorical matrix for defining vulnerability was based on a 3 × 3 matrix with vulnerability being categorized by nine possible combinations between the levels of response capacity and exposure. However, the initial completed BN model showed that the hidden variable “response capacity” with three categories was not distinguishable, while the elicited “exposure” levels with three categories were more distinctive. In our study, the information contained in the observed variables that define the “response capacity” was not strong enough to distinguish three categories. Based on the principle of parsimony for modeling, we decided to specify/model “response capacity” with two categories. Hence, the finalized conceptual model was modified with the vulnerability definition matrix to be a 2 × 3 one (two categories for response capacity and three categories for exposure). The vulnerability definition matrix implemented in our completed BN model is therefore a result of a compromise between our disciplinary understanding/intention and the technical possibility/limitation due to the information contained in the observed variables (35).

Observed variables were imported into the BN model. The category levels in each observed variable were defined *a priori* in the information table related to Figure 2. Because we had a very well-defined conceptual model, the BN model structure was manually specified by defining each hidden variables with various local naive Bayes models to explore the suitability of identifying and classifying the possible meaningful categorical groups with each hidden variable in the model. This was done initially for “response capacity” and “exposure.” Based on the principle of parsimony, the number of category levels for each hidden variable were determined arbitrarily.

The purpose of the FMD outbreak risk management decision-making system was to determine the vulnerability level as characterized by the producers' demographics variables. This was implemented by integrating the two individual naive Bayesian net (one for defining the response capacity and one for defining the exposure) into one BN model with each of the 12 demographics variables connected as a child node to both response capacity and exposure nodes. Essentially, this was equivalent to the merger of two naive Bayesian nets with those demographics variables as the common linkage.

To complete the BN model, the vulnerability node was added into the model that is categorically defined by “response capacity” and “exposure.” As shown in the Supplementary Material, this is a static representation of the interactive model. The model contains 55 variables/nodes, of which 41 variables are observed data and 14 are hidden variables. Hidden variables are distributed in four different layers according to our *a priori* knowledge regarding the relationship between the observed variables and the categories of exposure and response capacity and detailed in Table 1.

Since a BN model represents the joint distribution of all variables included in the model, any one (or more than one) variable(s) may be selected as a target variable (equivalent to the “response” variable in a regression model). Various inferential analyses can be performed by assuming different scenarios in terms of the “findings” of other variables. In this study, the primary statistical inference analysis was undertaken on the resulting BN model to investigate how, given one or more than observed variables/nodes, other variables/nodes changed. The focus of this analysis was to explore the interrelationships between vulnerability and producers' demographic variables and to identify key characteristics driving exposure, response capacity, and overall vulnerability. For example, by finding “evidence” in terms of the demographic variables (i.e., by assuming different farmers' profiles), the completed BN model allows us to investigate the vulnerability status and the nuances of its determining factors of response capacity and exposure. The BN model also allows us to examine the farmers profiles by assuming various vulnerability status (namely, different combinations of response capacity, and exposure levels). Although a BN model is not a solution to the universal problem of lack of representativeness of data regarding the study population, it is a logically consistent and systematic way to perform the what-if analysis regarding the FMD outbreak risk management.

The next step was to identify the relative influence of relevant variables on exposure, response capacity, and vulnerability using the Netica's built-in sensitivity analysis algorithm (28, 34). Responses to the observed variables were qualitatively categorized based on the potential contribution of the practice to the risk of exposure or response capacity of the producer and their enterprise in relation to FMD. The qualitative descriptors were determined based on biosecurity and EAD management literature and historical epidemiological evidence from previous FMD outbreaks (2, 17, 23). Based on the sensitivity analysis results, some variables in the initial model were removed from the final model due to their lack of influence (22, 29).

## Results

### Demographics and Husbandry Practice

Overall, postal and online responses were obtained from 497 sheep producers from five Australian States and Territories. A total of 448 responses were complete and included in the analysis. Table 2 provides a summary of the demographic and husbandry characteristics for participating producers. Most producers were over 50 years of age and third-generation farmers with more than 20 years of experience in sheep farming. Most properties were located in NSW and Victoria, with approximately half of the respondents running a mixed livestock enterprise.

**Table 2:** Demographic and husbandry characteristics of sheep producers participating in a cross-sectional study in 2017–2018.

**Characteristic**		***N* (%) respondents**
State		
	VIC	206 (46)
	NSW	197 (44)
	WA	21 (5)
	QLD	9 (2)
	SA	8 (2)
	NT	–
	ACT	–
	TAS	–
	NA	7
Age		
	18–25	7 (2)
	26–35	51 (11)
	36–50	122 (27)
	51–65	181 (40)
	66–80	81 (18)
	Over 80	6 (1)
Farming background		
	First generation	108 (24.3)
	Second generation	63 (14.2)
	Third generation	274 (61.6)
	NA	3
Years farming		
	<5	51 (11.5)
	5–10	50 (11.3)
	11–20	64 (14.4)
	More than 20	279 (62.8)
	NA	4
Production system		
	Sheep and other livestock	191 (50.1)
	Sheep and cropping	119 (31.2)
	Sheep only	66 (17.3)
	Sheep and other	5 (1.3)
	NA	67
Property size (ha)		
	Mean	2120.3
	Min–max	1.5–125,000.0
	Median	500.0
	5–95%	7.0–7570.5
Number of ewes		
	Mean	1,571
	Min–max	3–13,500
	Median	800
	5–95%	9–7,000

### Attitudes Toward Foot and Mouth Disease

Attitudes of producers toward FMD were investigated by the cross-sectional study. Overall concern about FMD was moderate, with a quarter of respondents (25.3%) reporting no concern and 22.6% reporting extreme levels of concern. The majority of respondents perceived little or no likelihood of an FMD outbreak occurring on their own property (92.8%) or region (80.3%). However, only 35.7% of producers thought there was little or no likelihood of an FMD outbreak somewhere in Australia. There was general agreement that an FMD outbreak would be extremely serious at all levels: on their property (78.3%), the region (73.6%), and the country (65.8%).

The study also asked producers about their level of confidence in identifying clinical signs of FMD in their animals, with only 12.8% reporting high levels of confidence and a third reporting little to no confidence.

#### Exposure-Related Practices

Among participating producers, there was good implementation of biosecurity practices in relation to incoming animals, with producers reporting that they always inspected new stock for disease (84.1%), used animal movement documentation (75.4%), inspected stock before purchase (71.3%), and isolated new animals (64.9%). However, implementation of other biosecurity measures, mainly in relation to feral animal control and visitors, was limited. Approximately a third of respondents reported regularly restricting access for visitors (32.3%) and having specific control plans for feral animals (29.6%). In addition, 53.6% of producers did not require visitors to follow biosecurity practices when visiting their property.

#### Response-Capacity-Related Practices

Practices used to define producer Response Capacity were mainly those related to inspection of animals, recognizing unusual signs of disease and reporting of these unusual signs to competent authorities or appropriate stakeholders.

The majority of respondents identified themselves as responsible for observing (94.6%), recognizing (79.3%), and reporting (91.0%) unusual signs of disease in their animals. In relation to routine inspection of animals, most producers reported visually inspecting their animals daily or weekly (90.0%) and almost half of respondents also reported a daily or weekly physical inspection of the animals (44.1%). Approximately a third of producers (27.7%) reported checking unwell animals every day. In addition, most producers (75.4%) reported checking their animals for disease prior to moving them off farm. Movement documentation when selling stock were used by 86.9% of respondents.

In relation to recognizing and reporting unusual signs of disease, nearly half of respondents reported that they usually (most of the times or always) report unusual signs of disease (49.0%), with 59.4% reported knowing who to call if they found unusual signs of disease in their animals. Most producers reported keeping records of animal health (96.9%) and stock movements (73.2%). Producers were also asked about their first three actions in the event of unusual signs of disease in their sheep, with calling a private veterinarian being the most commonly selected option (Table 3). However, a significant proportion of producers (31.1%) chose “Watch and wait” as one of the top three options. Furthermore, the questionnaire asked producers about their knowledge on subsidies to financially support the veterinary costs of reporting and diagnosis, with the majority (61.8%) being unaware of the existence of these subsidies.

**Table 3:** Ranking of actions in response to seeing unusual signs of disease in your sheep^*^.

**Response *n* (%)**	**First action**	**Second action**	**Third action**	**Not top 3**
Watch and wait	63 (15.3)	33 (8.0)	32 (7.8)	283 (68.8)
Do nothing	–	2 (0.5)	10 (2.4)	398 (97.0)
Call private vet	106 (25.9)	83 (20.2)	83 (20.2)	138 (33.7)
Call gov vet	61 (14.8)	64 (15.6)	57 (13.9)	229 (55.6)
Call disease hotline	9 (2.2)	16 (3.9)	29 (7.1)	356 (86.7)

* *These categories were selected from 11 response options, based on their impact on response capacity to a suspect FMD outbreak*.

Attitudes toward reporting were further investigated when producers were asked about their agreement on the effectiveness of reporting in preventing the spread of animal diseases, with the majority agreeing or strongly agreeing with the statement (68.3%).

### Bayesian Network Analysis

**Figure 3:**
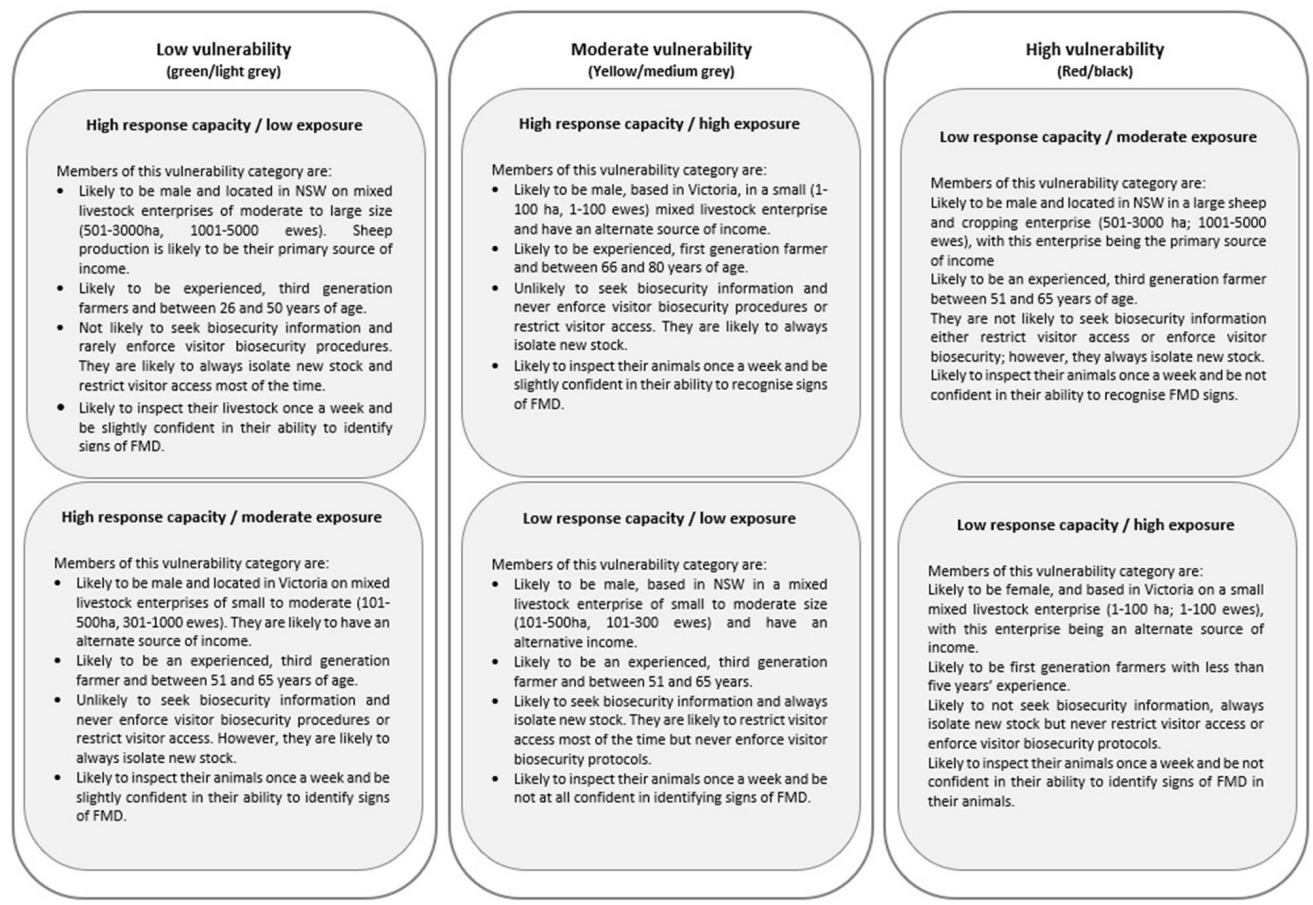
The three vulnerability states of Australian sheep producers according to response capacity and likelihood of exposure to foot and mouth disease (FMD).

Sheep producers participating in the study were categorized into different typologies of vulnerability to an FMD outbreak using the BN model and analysis. As a result of the analysis, six typologies were derived. The typologies are based on the likelihood of producers holding certain beliefs and adopting certain practices related to exposure and response capacity. The six typologies are summarized in Figure 3 and shown in full in the Supplementary Information.

#### Sensitivity Analysis

The sensitivity of the observed and hidden variables to affect the determination of the vulnerability status was estimated through the Netica's built-in sensitivity analysis procedure. The results are presented in Table 4 and can be interpreted as follows. The mutual information quantifies the “amount of information” obtained about the target variable “vulnerability” through observing the other random variables. Using sensitivity analysis, property size, number of ewes, visitor biosecurity, primary income, years of farming, state, restricted access, and education were identified as those variables with the greatest influence on respondent vulnerability.

**Table 4:** Bayesian network sensitivity analysis.

**Node**	Mutual information^*^	**Percent**
Vulnerability	2.51039	100
Exposure level	1.51051	60.2
Response capacity	0.99988	39.8
Inspection^**^	0.99984	39.8
Number of ewes	0.84204	33.5
Property size ha	0.81601	32.5
Recognizing^**^	0.70731	28.2
Attitude^**^	0.50905	20.3
State	0.41989	16.7
Years farming	0.41085	16.4
Primary income	0.35666	14.2
Restrict access	0.30847	12.3
Age	0.29445	11.7
Visitor biosecurity	0.27619	11.0

* *Mutual information (i.e., “entropy reduction”)—a measure of the dependence between two random variables, the changes in uncertainty of X due to knowing Y (36)*.

** *BN hidden variables*.

*** *Vulnerability was 100% explained by itself and the “Exposure Level” has the highest influence on defining vulnerability with 60.2% mutual information*.

*Note that the second highest influential variable was “Response capacity” (with 39.8% mutual information) and the two mutual information adding up to 100% (60.2 + 39.8 = 100) because the vulnerability status was deterministically defined by two sublevel hidden variables: response capacity and exposure level, as detailed in Figure 1*.

## Discussion

A BN model, populated by data from a cross-section survey of Australian sheep producers, has been used to enhance understanding of the vulnerability of producers to an FMD outbreak. This study found that sheep producers could be categorized into six risk-based typologies, based on two response capacity variables (high, low) and three exposure variables (low, moderate, high). This has resulted in a deeper understanding of the sheep industry in Australia and current biosecurity practices and exposure risks.

Overall, the risk vulnerability characteristics identified in the present study and in other similar studies strongly suggest that a one-size-fits-all approach to extension and implementation of surveillance activities across and within livestock industries may not be appropriate. Rather, more research into context-based drivers of, and barriers to, uptake of protective surveillance behavior at individual producer or risk-based cohort level is likely required. A wider perspective on institutional constraints to adoption of such behaviors is also required, reflected in the levels of trust in government veterinary agents.

The need for such a perspective is highlighted by considering the variation across sheep producers with predicted highest vulnerability to a FMD outbreak. According to the predictions of the BN model, these producers are moderate to large mixed cropping enterprises based in NSW (low response capacity, moderate exposure) and small, mixed livestock producers in Victoria (low response capacity, high exposure). The former cohort would most likely have animals of less individual worth and are older, more experienced farmers, with longer exposure to changes in agricultural governance over time (16). This may contribute to a high sense of self-efficacy and to a disconnection with agents of regulation, such as biosecurity officers and government veterinarians. The latter cohort with predicted high vulnerability are likely to have less experience and not come from a farming background. Their requirements for biosecurity information, animal health management skills, and partnerships with regulatory bodies will be different. Therefore, access to more specific information about producers based on their vulnerability profile may encourage more tailored communication and extension activities and will also inform more focused research in the future into adoption of and beliefs around biosecurity practices.

The BN model predicts that vulnerability of sheep producers increases as property size and ewe numbers decrease. This is supported by past research around smallholders and smaller producers. Hernandez-Jover et al. (37) found that smallholders with sheep were associated with a decreased engagement around surveillance activities such as frequency of animal inspection and use of veterinary services. This finding is in contrast to a BN model analysis looking at the vulnerability of Australian beef producers to an FMD outbreak (22), where the model indicated that larger beef producers are more likely to be categorized as highly vulnerable. The findings from the sheep produce study confirm the need for greater investment in relationships and network building with smallholders, to encourage interactions between private/government veterinarians and landholders with small numbers of sheep. This is particularly significant given the mild and transient nature of FMD signs in sheep.

When considering the top three actions that producers would undertake in response to unusual signs of disease, the majority of sheep producers reported that they would not watch and wait (68.8%) or do nothing (97.0%). When reflecting on the role that private veterinarians might play in an early response to unusual signs of animal disease, two-thirds of respondents reported that they would contact their private veterinarian (66.3%), with just less than half reporting that they would contact their government veterinarian (44.4%). When examining the role of trust in the predicted vulnerability profiles, the likelihood of sheep producers having high levels of trust in both private and government veterinarians decreases slightly as vulnerability increases. This is in contrast to the reported early response of Australian beef producers to unusual signs of disease (22). The majority of those beef producers (83.6%) reported that calling their private veterinarian would be one of their top three actions, with 43.8% contacting their government veterinarian. However, the likelihood of producers having high levels of trust both private and government veterinarians increased as vulnerability increased. These variations again reflect the need for a wide systems-based approach that can examine the actions and beliefs from multiple stakeholders within the industries and government agencies (14). The importance of including social and psychological research findings into biosecurity projects to examine institutional and personal barriers and drivers that impact Australia's vulnerability to an EAD should not be underestimated.

While trust in veterinary services is recognized as important for adoption of biosecurity messages (16, 38), these results, including the low sensitivity of the model to producer trust in veterinarians (Table 4), need clarification. Further research is required for a deeper understanding of the role that trust plays in the producer–veterinarian relationship. However, the low sensitivity to trust found by the BN model signals the need to prioritize relationship building, possibly over regulation, and to consider strengthening relationships when allocating future surveillance resources, including location and selection of government veterinarians (14, 16, 39, 40) and training of private veterinarians (41, 42).

The BN model approach cannot only be used to understand vulnerability but also to reflect on the distribution and utilization of current surveillance resources. When considering the disease hotline as part of the early response system available to livestock producers, sheep producers who were part of this study were very unlikely to use the disease hotline if they saw anything unusual in their animals, regardless of location or vulnerability level. This reported low uptake is also replicated in the study of beef producer vulnerability (22) and goat producers (29) and does suggest that adoption of this resource as a reporting and support tool might benefit from further examination of barriers to adoption and subsequent strengthening of its capability and usefulness to producers.

Recent research around adoption of biosecurity behaviors by livestock producers has highlighted the importance of focusing on specific behaviors when considering behavior change and adoption of new practices (43, 44). In this study, nearly three-fourths of respondents reported using the appropriate movement documentation when buying or selling stock. This suggests that there is an opportunity to use animal movement documentation as a gateway to behavior adoption of other desirable biosecurity practices. This might include a checklist embedded in the existing documentation as a prompt or a further breakdown of the desirable practices in order to remove barriers to uptake.

The sensitivity analysis of the BN model indicates that the input factors that most strongly influence sheep producer vulnerability are the exposure variables of restricting visitor access and enforcing visitor biosecurity practices. This finding also highlights the importance of deeper examination into the barriers to adoption of these practices and consideration of how these practices are communicated about (22, 45, 46). Results from the BN analysis suggests that exposure variables have more influence over a producer's vulnerability than response capacity. Future research, including evaluation of the models' predictive capacity using external data from independent on-farm vulnerability assessments will explore the robustness of the assumptions made in the development of the vulnerability framework. This work will also explore the usefulness of the concepts of exposure and response capacity to inform strengthening of Australia's preparedness for an EAD outbreak.

Vulnerability of the Australian sheep industry can be analyzed across geographic regions using the BN model to reflect on existing jurisdictional surveillance systems. Previous research found that the eastern and southern regions of Australia have a higher likelihood of entry, establishment, and spread of FMD (5). The BN analysis found that sheep producers with the highest vulnerability to an FMD outbreak are likely to be located in NSW (88.6% low response capacity, moderate exposure) and Victoria (88.4% low response capacity, high exposure). The overlap between these findings and that of East et al. (5) should inform development and evaluation of future risk-based surveillance strategies. East et al. (5) concluded that there is limited opportunity for improving current surveillance strategies based on geographic risk-based approaches because current surveillance activities are already focused in the areas of greatest risk. Our study strengthens these findings and allows for a deeper examination of actual on-farm practices. This approach can inform how existing surveillance strategies may be enhanced by focusing on the activities themselves. The model also predicts the likelihood of sheep producers in the study always/most of the time enforcing visitor biosecurity practices, showing increases from Victorian producers (12.3%), to NSW producers (17.3%), and Queensland producers (24.8%). The same gradient is observed when considering restricting visitor access: Victoria (27.3%), New South Wales (35.9%), and Queensland (44.6%). This comparison may provide an opportunity to examine the efficacy of any existing extension activities that focuses on these practices and facilitate interjurisdictional sharing and consultation around communication strategies and extension approaches.

The need to confine response capacity to two categories also needs reflection. This may indicate that the concept of response capacity needs to be clarified to avoid missing more nuanced data. It may also be that the questions used to collect the response capacity data may need revision. As part of the evaluation process, these issues will be addressed.

While these results are useful in providing insights into vulnerability characteristics of sheep producers, the limited representativeness of the sample in reflecting the total population of Australian sheep producers is a constraint of this study. For example, Western Australian sheep producers were underrepresented in the data collected, as were South Australian sheep producers. The south western coast of Western Australia was identified by East et al. (5) as an area of risk for introduction, establishment, and spread of FMD. This lack of representativeness can be a common limitation of self-report data. More targeted and tightly enforced data collection methods with subsequent analysis using the BN model could facilitate wider consideration of the Australian sheep industry's vulnerability to an incursion of FMD, but this approach is also not without its limitations. However, the benefits of using the BN approach means that the exploratory findings from this model are still useful to inform potential policy considerations and future directions of research, despite not being intended to be generalizable. New data can also be added at any stage and the model updated.

Future work may also include examination of vulnerability of different enterprises on the same farm, when producers farm more than one species. Another factor to consider is the capacity of the model to capture the impact on vulnerability, of how different species of livestock react to FMD, with sheep clinical signs being less apparent that cattle and pigs (18) and therefore more likely to go unnoticed for longer.

A further point to consider when interpreting these results is that the link to the questionnaire was distributed through emails, newsletters, social media, etc. Thus, there may be sampling bias in utilizing a convenience sample (47), and response rate cannot be determined.

The limitations of a BN methodological approach to examine Australian sheep producer vulnerability are defined primarily by the number and nature of the variables. The selection of variables is not exhaustive, and their inclusion needs to be considered in light of historical and current epidemiological data. The advantages of the BN approach is its capacity to incorporate new data and the resultant ongoing model validation process. Continuing research in the use of BN models and increasing software capacity ensures that the limitations around variables is becoming less significant.

Evaluation of the model is being undertaken with input of new producer data to test the model's predictions against on-farm assessments. This process will also progress consideration of the usefulness of the concept of vulnerability as a tool to strengthen Australian sheep producers' preparedness for a possible FMD outbreak.

## Conclusion

The present study used a BN model to interrogate questionnaire data that reflects producer practices and beliefs around surveillance and biosecurity and develop vulnerability typologies of Australian sheep producers. Examination of the typologies and the practices and beliefs of producers increases understanding around how to enhance the capacity of biosecurity and surveillance resources and identify opportunities for improving Australia's preparedness for any future EAD incursion.

The results from this study highlight that more work is needed to understand drivers of and barriers to sheep producer uptake of biosecurity messages, so that risk management and communication strategies are appropriate to the enterprise and delivered in a way that facilitates adoption. This may include revising current biosecurity protocols for the Australian sheep industry to ensure that barriers to adoption are addressed and embedding biosecurity messages in existing tools such as animal movement documentation that appear to be more readily adopted.

The BN model approach has afforded a nuanced, holistic perspective through which to consider sheep producer vulnerability to a FMD outbreak and the development of a tool with the potential to support risk-based allocation of resources for animal disease surveillance in Australia.

## Data Availability Statement

The original contributions presented in the study are included in the article/Supplementary Material, further inquiries can be directed to the corresponding author/s.

## Ethics Statement

The studies involving human participants were reviewed and approved by Charles Sturt University Human Research Ethics Committee. Written informed consent for participation was not required for this study in accordance with the national legislation and the institutional requirements.

## Author Contributions

JM, YM, LH, BL, HK, AM, RW, and MH-J made significant contributions to the development of the questionnaire. JM, LH, and MH-J were responsible for the distribution of the questionnaire and the descriptive data analysis. GX developed the BN model, with contributions from JM, LH, and MH-J to the framework. BN analysis was undertaken by JM. JM was responsible for the manuscript with significant contributions from YM, LH, BL, HK, AM, GX, RW, and MH-J. All authors contributed to the article and approved the submitted version.

## Conflict of Interest

The authors declare that the research was conducted in the absence of any commercial or financial relationships that could be construed as a potential conflict of interest.

## References

[1] Department of Agriculture Water Resources. Farm Survey Data. (2019). ABARES. Available online at: https://www.agriculture.gov.au/abares/research-topics/surveys/farm-survey-data

[2] Matthews K. A Review of Australia's Preparedness for the Threat of Foot-and-Mouth Disease. Canberra: Department of Agriculture, Fisheries and Forestry (2011).

[3] Singh D. New infectious diseases will continue to emerge. Br Med J. (2004) 328:186. 10.1136/bmj.328.7433.186-c 14739176 PMC1140655

[4] DAFF Biosecurity. Reform of Australia's Biosecurity System: New Biosecurity Legislation. Canberra: Fisheries and Forestry Department of Agriculture (2012).

[5] East IJ Wicks RM Martin PAJ Sergeant ESG Randall LA Garner MG. Use of a multi-criteria analysis framework to inform the design of risk based general surveillance systems for animal disease in Australia. Prev Vet Med. (2013) 112:230–47. 10.1016/j.prevetmed.2013.09.012 24125696

[6] Barclay E. Local Community Preparedness for an Emergency Animal Disease Outbreak. Canberra, ACT: Institute for Rural Futures (2005).

[7] Buetre B Wicks S Kruger H Millist N Yainshet A Garner G. Potential Socio-Economic Impacts of an Outbreak of Foot-and Mouth-Disease in Australia. Canberra: ABARECanberra S (2013).

[8] Stärk DKC Gertraud R Jorge H Lea K Klemens F Morris RS. Concepts for risk-based surveillance in the field of veterinary medicine and veterinary public health: Review of current approaches. BMC Health Serv Res. (2006) 6:20–20. 10.1186/1472-6963-6-20 16507106 PMC1409776

[9] Garner MG East IJ Kompas T Ha PV Roche SE Nguyen HT. Comparison of alternatives to passive surveillance to detect foot and mouth disease incursions in Victoria, Australia. Prev Vet Med. (2016) 128:78–86. 10.1016/j.prevetmed.2016.04.009 27237393

[10] Higgins V Melanie B Marta H-J Luzia R Connar M. Devolved responsibility and on-farm biosecurity: practices of biosecure farming care in livestock production. Sociol Rural. (2018) 58:20–39. 10.1111/soru.12155

[11] Nairn ME Allen PG Inglis AR Tanner C. Australian Quarantine. Canberra, ACT: Department of Primary Industries and Energy (1996).

[12] Palmer S Sully M Fozdar F. Farmers, animal disease reporting and the effect of trust: A study of West Australian sheep and cattle farmers. Rural Soc. (2009) 19:32–48. 10.5172/rsj.351.19.1.32

[13] Higgins V Bryant M Hernández-Jover M McShane C Rast L. Harmonising devolved responsibility for biosecurity governance: the challenge of competing institutional logics. Environ Plann. (2016) 48:1133–51. 10.1177/0308518X16633471

[14] Maru Y Hernandez-Jover M Loechel B Manyweathers J Mankad A Hayes L. Towards Piloting Producer-Led Partnerships for Surveillance: Learning From the Current State of Animal Health Surveillance and Partnership Inititaives. Canberra, ACT: CSIRO (2017).

[15] Taylor M Dhand NK Lee A. Farm Biosecurity Attitudes and Practices: Factors Influencing the Sheep Industry. Wagga Wagga: NSW: Meat and Livestock Australia (2011).

[16] Palmer S Fozdar F Sully M. The effect of trust on west australian farmers' responses to infectious livestock diseases. Soc Rural. (2009) 49:360–74. 10.1111/j.1467-9523.2009.00495.x

[17] Bourn J. The 2001 Outbreak of Foot and Mouth Disease. London: Bourn - National Audit Office (2002).

[18] Animal Health Australia. Disease strategy: Foot and Mouth Disease (version 3.4). Australian Veterinary Emergency Plan (AUSVETPLAN). Canberra, ACT: Agricultural Ministers Forum (2014).

[19] East IJ Martin PAJ Langstaff I Iglesias RM Sergeant ESG Garner MG. Assessing the delay to detection and the size of the outbreak at the time of detection of incursions of foot and mouth disease in Australia. Prev Vet Med. (2016) 123:1–11. 10.1016/j.prevetmed.2015.12.005 26718055

[20] Garner MG Bombarderi N Cozens M Conway ML Wright T Paskin R. Estimating resource requirements to staff a response to a medium to large outbreak of foot and mouth disease in Australia. Transb Emerg Dis. (2016) 63:e109–21. 10.1111/tbed.12239 24894407

[21] Martin PAJ Langstaff I Iglesias RM East IJ Sergeant ESG Garner MG. Assessing the efficacy of general surveillance for detection of incursions of livestock diseases in Australia. Prev Vet Med. (2015) 121:215–30. 10.1016/j.prevetmed.2015.06.017 26255687

[22] Manyweathers J Yiheyis M Lynne H Barton L Heleen K Aditi M. Understanding the vulnerability of beef producers in Australia to an FMD outbreak using a Bayesian Network predictive model. Prev Vet Med. (2020) 175:104872. 10.1016/j.prevetmed.2019.104872 31981953

[23] Hernández-Jover M Nicole S Patricia Holyoake K Jenny-Ann Toribio LML Peter Anthony Julian M. A comparative assessment of the risks of introduction and spread of foot-and-mouth disease among different pig sectors in Australia. Front Vet Sci. (2016) 3:85. 10.3389/fvets.2016.00085 27713881 PMC5031773

[24] Hernández-Jover M Gilmour J Schembri N Sysak T Holyoake PK Beilin R. Use of stakeholder analysis to inform risk communication and extension strategies for improved biosecurity amongst small-scale pig producers. Prev Vet Med. (2012) 104:258–270. 10.1016/j.prevetmed.2011.12.006 22227304

[25] Davis M Niamh S Paul F. Compliant, complacent or panicked? Investigating the problematisation of the Australian general public in pandemic influenza control. Soc Sci Med. (2011) 72:912–8. 10.1016/j.socscimed.2011.01.016 21349624

[26] Schembri N Hernandez-Jover M Toribio JALML Holyoake PK. On-farm characteristics and biosecurity protocols for small-scale swine producers in eastern Australia. Prev Vet Med. (2015) 118:104–16. 10.1016/j.prevetmed.2014.11.008 25433716

[27] Manyweathers J Field H Jordan D Longnecker N Agho K Smith C. Risk Mitigation of Emerging Zoonoses: Hendra Virus and Non-Vaccinating Horse Owners. Transb Emerg Dis. (2017) 64:1898–1911. 10.1111/tbed.12588 28054443

[28] Kjærulff U Madsen AL. Bayesian networks and influence diagrams: a guide to construction and analysis. J Am Stat Assoc. (2008) 12:1273.

[29] Manyweathers J Maru Y Hayes L Loechel B Kruger H Mankad A. The goat industry in Australia: using Bayesian network analysis to understand vulnerability to a foot and mouth disease outbreak. Prev Vet Med. (2021) 187:105236. 10.1016/j.prevetmed.2020.105236 33385617

[30] Nelson R Kokic P Crimp S Martin P Meinke H Howden SM. The vulnerability of Australian rural communities to climate variability and change: Part II—Integrating impacts with adaptive capacity. Environ Sci Policy. (2010) 13:18–27. 10.1016/j.envsci.2009.09.007

[31] Korb KB Nicholason AE. Bayesian Artifical Intelligence. New York, NY: CRC Press, Taylor and Francis group (2011).

[32] Australian Government. A Beginners Guide to Bayesian Network Modelling for Integrated Catchment Management. Canberra, ACT: Landscape Logic (2009).

[33] Pearl J. Probabilistic Reasoning in Intelligent Systems. San Mateo, CA: Morgan Kaufmann (1988).

[34] Norsys Software Corp. Netica [version 6.5]. (2018). Available online at: https://www.norsys.com/

[35] Darwiche A. Modeling and Reasoning With Bayesian Networks. Cambridge: Cambridge University Press (2009). 10.1017/CBO9780511811357

[36] Gomez-Villegas MA Main P Viviani P. Sensitivity to evidence in Gaussian Bayesian networks using mutual information. Inf Sci. (2014) 275:115–26. 10.1016/j.ins.2014.02.025

[37] Hernandez-Jover M Lynne H Robert W Luzia R Jenny-Ann Toribio LML. Animal health management practices among smallholder livestock producers in australia and their contribution to the surveillance system. Front Vet Sci. (2019) 6:191. 10.3389/fvets.2019.00191 31275950 PMC6591531

[38] Brennan ML Christley RM. Cattle producers' perceptions of biosecurity. BMC Vet Res. (2013) 9:1–8. 10.1186/1746-6148-9-71 23574789 PMC3626881

[39] Cairns G de Andrade M MacDonald L. Reputation, relationships, risk communication, and the role of trust in the prevention and control of communicable disease: a review. J Health Commun. (2013) 18:1550–65. 10.1080/10810730.2013.840696 24298887

[40] Manyweathers J Field H Longnecker N Agho K Smith C Taylor M. Why won't they just vaccinate? Horse owner risk perception and uptake of the Hendra virus vaccine. BMC Vet Res. (2017) 13:1–12. 10.1186/s12917-017-1006-7 28407738 PMC5390447

[41] Svensson C Emanuelson U Bard AM Forsberg L Wickström H Reyher KK. Communication styles of Swedish veterinarians involved in dairy herd health management: A motivational interviewing perspective. J Dairy Sci. (2019) 102:10173–85. 10.3168/jds.2018-15731 31521349

[42] Bard AM David Main CJ Anne Haase M Helen Whay R Emma Roe J Kristen Reyher K. The future of veterinary communication: Partnership or persuasion? A qualitative investigation of veterinary communication in the pursuit of client behaviour change. PLoS ONE. (2017) 12:e0171380. 10.1371/journal.pone.0171380 28257511 PMC5336212

[43] Richens IF Houdmont J Wapenaar W Shortall O Kaler JH O'connor. Application of multiple behaviour change models to identify determinants of farmers' biosecurity attitudes and behaviours. Prev Vet Med. (2018) 155:61–74. 10.1016/j.prevetmed.2018.04.010 29786526

[44] Wright BK Bradley Jorgensen S Liam Smith DG. Understanding the biosecurity monitoring and reporting intentions of livestock producers: identifying opportunities for behaviour change. Prev Vet Med. (2018) 157:142–51. 10.1016/j.prevetmed.2018.07.007 30086842

[45] Bard AM David M Emma R Anne H Helen Rebecca W Kristen Reyher K. To change or not to change? Veterinarian and farmer perceptions of relational factors influencing the enactment of veterinary advice on dairy farms in the United Kingdom. J Dairy Sci. (2019) 102:10379–94. 10.3168/jds.2019-16364 31447158

[46] Svensson C Lind N Reyher KK Bard AM Emanuelson U. Trust, feasibility, and priorities influence Swedish dairy farmers' adherence and nonadherence to veterinary advice. J Dairy Sci. (2019) 102:10360–68. 10.3168/jds.2019-16470 31495620

[47] Dillman DA. Internet, Phone, Mail, and Mixed-Mode Surveys: The Tailored Design Method. Hoboken, NJ: Wiley (2014).

