# Supplementary material for: Using a Bayesian Network Predictive Model to Understand Vulnerability of Australian Sheep Producers to a Foot and Mouth Disease Outbreak
Source: Front Vet Sci. 2021 Jun 11;8:668679. doi: 10.3389/fvets.2021.668679 (PMC8226010; doi:10.3389/fvets.2021.668679)
Supplement: Supplementary file 1 [file Data_Sheet_1.PDF]

Supporting Information: A typology of demographics of Australian sheep producer states of vulnerability to an FMD incursion, based on their exposure risk and response capacity.

Vulnerability		Low		Moderate		High	
Category (%)	All	High response capacity/low exposure	High response capacity/moderate exposure	High response capacity/High exposure	Low response capacity/low exposure	Low response capacity/Moderate exposure	Low response capacity/high exposure
State	Vic (46.6)<br>NSW (44.6)<br>WA (4.8)<br>QLD (2.1)<br>SA (1.9)	NSW (44.3)<br>Vic (25.9)<br>SA (11.8)<br>WA (10.9)<br>QLD (7.2)	Vic (67.7)<br>NSW (31.2)<br>QLD (1.1)<br>WA (0.0)<br>SA (0.0)	Vic (91.5)<br>NSW (4.5)<br>WA (4.0)<br>QLD (0.0)<br>SA (0.0)	NSW (47.8)<br>Vic (43.5)<br>WA (4.7)<br>QLD (4.0)<br>SA (0.0)	NSW (88.6)<br>WA (6.7)<br>Vic (4.7)<br>QLD (0.0)<br>SA (0.0)	Vic (88.4)<br>NSW (9.2)<br>WA (2.4)<br>QLD (0.0)<br>SA (0.0)
Production system	Sheep and other livestock (50.9)<br>Sheep and crops (30.0)<br>Sheep only (17.8)<br>Other (1.3)	Sheep and other livestock (45.7)<br>Sheep and crops (33.9)<br>Sheep only (18.9)<br>Other (1.6)	Sheep other livestock (50.7)<br>Sheep and crops (35.6)<br>Sheep only (13.7)<br>Other (0.0)	Sheep and other livestock (67.8)<br>Sheep only (28.9)<br>Sheep and crops (0.0)<br>Other (3.3)	Sheep other livestock (63.3)<br>Sheep only (26.8)<br>Sheep and crops (5.6)<br>Other (4.3)	Sheep and crops (54.7)<br>Sheep other livestock (36.6)<br>Sheep only (8.7)<br>Other (0.0)	Sheep other livestock (55.5)<br>Sheep and crops (24.0)<br>Sheep only (20.4)<br>Other (0.0)
Property size (ha)	501-3000 (38.9)<br>1-100 (24.7)<br>101-500 (22.9)<br>>3001 (13.5)	501-3000 (46.3)<br>>3001 (41.6)<br>101-500 (10.5)<br>1-100 (1.6)	101-500 (53.4)<br>501-3000 (41.5)<br>>3001 (2.9)<br>1-100 (2.3)	1-100 (87.2)<br>101-500 (12.8)<br>501-3000 (0.0)<br>>3001 (0.0)	101-500 (42.1)<br>1-100 (31.4)<br>501-3000 (16.4)<br>>3001 (10.1)	501-3000 (81.3)<br>>3001 (18.7)<br>1-100 (0.0)<br>101-500 (0.0)	1-100 (94.3)<br>101-500 (5.7)<br>501-3000 (0.0)<br>>3001 (0.0)
Number of ewes	1001-5000 (37.2)<br>301-1000 (22.5)<br>1-100 (20.7)<br>101-300 (11.1)<br>> 5000 (8.6)	1001-5000 (53.3)<br>> 5000 (27.2)<br>301-1000 (17.9)<br>101-300 (1.59)<br>1-100 (0.0)	301-1000 (55.3)<br>1001-5000 (37.9)<br>101-300 (3.9)<br>> 5000 (2.8)<br>1-100 (0.0)	1-100 (79.1)<br>101-300 (20.9)<br>301-1000 (0.0)<br>1001 – 5000 (0.0)<br>> 5000 (0.0)	101-300 (38.5)<br>1-100 (21.8)<br>301-1000 (19.1)<br>1001-5000 (19.0)<br>> 5000 (1.5)	1001-5000 (70.6)<br>301-1000 (15.6)<br>> 5000 (13.9)<br>101-300 (0.0)<br>1-100 (0.0)	1-100 (86.9)<br>101-300 (13.1)<br>301-1000 (0.0)<br>1001 – 5000 (0.0)<br>> 5000 (0.0)
Years farming	>20 (62.2)<br>11-20 (14.6)<br>< 5 (11.9)<br>5-10 (11.3)	>20 (44.9)<br>11-20 (37.0)<br>5-10 (14.7)<br>< 5 (3.5)	>20 (69.7)<br>5-10 (12.6)<br>11-20 (12.1)<br>< 5 (5.6)	>20 (44.4)<br>5-10 (24.1)<br>< 5 (20.9)<br>11-20 (10.5)	>20 (65.8)<br>11-20 (19.8)<br>< 5 (7.6)<br>5-10 (6.8)	>20 (99.2)<br>< 5 (0.8)<br>11-20 (0.0)<br>5-10 (0.0)	< 5 (62.6)<br>5-10 (23.8)<br>11-20 (13.6)<br>>20 (0.0)
Farming background (Generation)	Third (61.5)<br>First (24.7)<br>Second (13.9)	Third (82.1)<br>Second (8.5)<br>First (9.4)	Third (68.7)<br>First (17.6)<br>Second (13.7)	First (41.4)<br>Third (36.6)<br>Second (22.0)	Third (54.8)<br>First (25.7)<br>Second (19.5)	Third (77.4)<br>Second (14.3)<br>First (8.2)	First (83.2)<br>Third (12.2)<br>Second (4.6)
Number of workers on farm	1-5 (78.8)<br>None (18.9)	1-5 (94.1)<br>6-10 (5.9)	1-5 (73.6)<br>None (26.4)	1-5 (54.2)<br>None (45.8)	1-5 (81.1)<br>None (17.4)	1-5 (89.1)<br>6-10 (2.2)	1-5 (63.8)<br>None (33.6)

	6-10 (1.5)<br>>10 (0.8)	None (0.0)<br>>10 (0.0)	6-10 (0.0)<br>>10 (0.0)	6-10 (0.0)<br>>10 (0.0)	>10 (1.5)<br>6-10 (0.0)	None (7.7)<br>>10 (1.1)	>10 (2.7)<br>6-10 (0.0)
Primary income	No (58.7)<br>Yes (41.3)	Yes (60.1)<br>No (39.9)	No (73.2)<br>Yes (26.8)	No (97.5)<br>Yes (2.5)	No (74.6)<br>Yes (25.4)	Yes (88.8)<br>No (11.2)	No (100)<br>Yes (0.0)
Age (years)	51-65 (40.2)<br>36-50 (27.8)<br>66-80 (17.4)<br>26-35 (11.6)<br>18-25 (1.6)<br>Over 80 (1.3)	36-50 (34.5)<br>26-35 (34.2)<br>51-65 (23.7)<br>18-25 (6.6)<br>66-80 (0.0)<br>Over 80 (0.9)	51-65 (30.2)<br>36-50 (26.5)<br>26-35 (17.6)<br>66-80 (14.2)<br>18-25 (1.3)<br>Over 80 (2.0)	66-80 (45.8)<br>51-65 (30.2)<br>36-50 (12.0)<br>26-35 (12.0)<br>18-25 (0.0)<br>Over 80 (0.0)	51-65 (50.7)<br>66-80 (27.3)<br>36-50 (17.1)<br>Over 80 (1.8)<br>26-35 (1.7)<br>18-25 (1.4)	51-65 (54.9)<br>36-50 (20.2)<br>66-80 (20.9)<br>26-35 (2.1)<br>Over 80 (1.9)<br>18-25 (0.0)	36-50 (70.1)<br>51-65 (29.9)<br>66-80 (0.0)<br>26-35 (0.0)<br>18-25 (0.0)<br>Over 80 (0.0)
Gender	Male (74.2)<br>Female (25.8)	Male (56.4)<br>Female (43.6)	Male (89.1)<br>Female (10.9)	Male (65.5)<br>Female (34.5)	Male (67.1)<br>Female (32.9)	Male (92.1)<br>Female (7.9)	Female (51.9)<br>Male (48.1)
Education	Degree (26.7)<br>Tafe (26.6)<br>Year 10 (18.4)<br>Year 12 (15.7)<br>Postgrad (10.7)<br>Primary (1.8)	Degree (53.8)<br>Year 12 (22.1)<br>Postgrad (14.4)<br>Tafe (9.7)<br>Year 10 (0.0)<br>Primary (0.0)	Tafe (34.5)<br>Year 10 (28.2)<br>Degree (16.8)<br>Year 12 (10.3)<br>Postgrad (5.4)<br>Primary (4.9)	Degree (39.8)<br>Postgrad (21.4)<br>Year 12 (18.5)<br>Tafe (8.4)<br>Year 10 (7.9)<br>Primary (4.1)	Tafe (32.4)<br>Year 10 (29.8)<br>Degree (19.3)<br>Postgrad (14.5)<br>Year 12 (4.0)<br>Primary (0.0)	Tafe (32.2)<br>Year 12 (24.9)<br>Degree (20.5)<br>Year 10 (18.0)<br>Postgrad (3.4)<br>Primary (1.0)	Tafe (31.7)<br>Degree (19.7)<br>Year 10 (18.6)<br>Postgrad (17.0)<br>Year 12 (12.9)<br>Primary (0.0)
Seek Biosecurity information	No (67.1)<br>Yes (32.9)	No (59.5)<br>Yes (40.5)	No (95.2)<br>Yes (4.8)	No (77.9)<br>Yes (22.1)	Yes (62.9)<br>No (37.1)	No (68.5)<br>Yes (31.5)	No (51.5)<br>Yes (48.5)
Isolate new stock	Always (64.4)<br>Most of the time (13.8)<br>Never (10.9)<br>Occasionally (6.7)<br>Rarely (4.2)	Always (79.9)<br>Most of the time (11.8)<br>Never (3.8)<br>Rarely (3.8)<br>Occasionally (0.7)	Always (64.1)<br>Most of the time (17.0)<br>Occasionally (9.7)<br>Rarely (4.6)<br>Never (4.5)	Always (40.5)<br>Never (36.7)<br>Occasionally (9.5)<br>Most of the time (9.4)<br>Rarely (4.0)	Always (79.9)<br>Most of the time (11.8)<br>Never (3.8)<br>Rarely (3.8)<br>Occasionally (0.7)	Always (64.1)<br>Most of the time (17.0)<br>Occasionally (9.7)<br>Rarely (4.6)<br>Never (4.5)	Always (40.5)<br>Never (36.7)<br>Occasionally (9.5)<br>Most of the time (9.4)<br>Rarely (4.0)
Restrict visitor access	Never (31.7)<br>Rarely (21.0)<br>Most of the time (18.3)<br>Occasionally (14.7)<br>Always (14.4)	Most of the time (32.8)<br>Always (31.6)<br>Rarely (17.9)<br>Occasionally (14.6)<br>Never (3.1)	Never (38.5)<br>Rarely (28.4)<br>Occasionally (19.6)<br>Most of the time (10.9)<br>Always (2.5)	Never (61.8)<br>Always (14.0)<br>Most of the time (12.0)<br>Rarely (8.4)<br>Occasionally (3.8)	Most of the time (32.8)<br>Always (31.6)<br>Rarely (17.9)<br>Occasionally (14.6)<br>Never (3.1)	Never (38.5)<br>Rarely (28.4)<br>Occasionally (19.6)<br>Most of the time (10.9)<br>Always (2.5)	Never (61.8)<br>Always (14.0)<br>Most of the time (12.0)<br>Rarely (8.4)<br>Occasionally (3.8)
Inforce visitor biosecurity procedures	Never (53.7)<br>Rarely (18.6)<br>Occasionally (12.2)<br>Most of the time (9.7)<br>Always (5.9)	Rarely (25.9)<br>Never (20.9)<br>Most of the time (20.5)<br>Always (17.8)<br>Occasionally (14.9)	Never (62.0)<br>Rarely (20.0)<br>Occasionally (14.4)<br>Most of the time (3.7)<br>Always (0.0)	Never (87.0)<br>Most of the time (6.3)<br>Rarely (4.0)<br>Occasionally (2.7)<br>Always (0.0)	Rarely (25.9)<br>Never (20.9)<br>Most of the time (20.5)<br>Always (17.8)<br>Occasionally (14.9)	Never (62.0)<br>Rarely (20.0)<br>Occasionally (14.4)<br>Most of the time (3.7)<br>Always 0.0)	Never (87.0)<br>Most of the time (6.3)<br>Rarely (4.0)<br>Occasionally (2.7)<br>Always (0.0)

Frequency of visual animal checks	Once a week (54.3)<br>Every day (35.7)<br>Few times year (4.4)<br>Once a month(4.4)<br>Once a year (1.2)<br>Never (0.0)	Once a week (52.8)<br>Every day (36.6)<br>Few times year (5.1)<br>Once a month(4.2)<br>Once a year (1.4)<br>Never (0.0)	Once a week (52.8)<br>Every day (36.6)<br>Few times year (5.1)<br>Once a month(4.2)<br>Once a year (1.4)<br>Never (0.0)	Once a week (52.8)<br>Every day (36.6)<br>Few times year (5.1)<br>Once a month(4.2)<br>Once a year (1.4)<br>Never (0.0)	Once a week (55.9)<br>Every day (34.9)<br>Once a month(4.6)<br>Few times year (3.7)<br>Once a year (0.9)<br>Never (0.0)	Once a week (55.9)<br>Every day (34.9)<br>Once a month(4.6)<br>Few times year (3.7)<br>Once a year (0.9)<br>Never (0.0)	Once a week (55.9)<br>Every day (34.9)<br>Once a month(4.6)<br>Few times year (3.7)<br>Once a year (0.9)<br>Never (0.0)
Confidence in identifying sighs of FMD	Not at all (37.4)<br>Slightly (29.8)<br>Moderately (19.7)<br>Very (10.0)<br>Extremely (3.1)	Slightly (38.9)<br>Not at all (31.9)<br>Moderately (14.7)<br>Very (10.5)<br>Extremely (3.9)	Slightly (38.9)<br>Not at all (31.9)<br>Moderately (14.7)<br>Very (10.5)<br>Extremely (3.9)	Slightly (38.9)<br>Not at all (31.9)<br>Moderately (14.7)<br>Very (10.5)<br>Extremely (3.9)	Not at all (42.8)<br>Moderately (24.4)<br>Slightly (20.9)<br>Very (9.6)<br>Extremely (2.3)	Not at all (42.8)<br>Moderately (24.4)<br>Slightly (20.9)<br>Very (9.6)<br>Extremely (2.3)	Not at all (42.8)<br>Moderately (24.4)<br>Slightly (20.9)<br>Very (9.6)<br>Extremely (2.3)
